# [Retracted] Carnosol inhibits osteoclastogenesis *in vivo* and *in vitro* by blocking the RANKL‑induced NF‑κB signaling pathway

**DOI:** 10.3892/mmr.2024.13369

**Published:** 2024-10-18

**Authors:** Pan Cai, Shichang Yan, Yan Lu, Xiaoxiao Zhou, Xiuhui Wang, Minghui Wang, Zhifeng Yin

Mol Med Rep 26: 225, 2022; DOI: 10.3892/mmr.2022.12741

Following the publication of this paper, it was drawn to the Editor's attention by a concerned reader that one of the data panels in Fig. 3A on p. 6, showing how carnosol inhibits RANKL-induced osteoclastogenesis in the early stage of differentiation, was strikingly similar to data that had already been submitted for publication in another article in the journal *Annals of Translational Medicine* written by different authors at different research institutes.

Owing to the fact that the contentious data in the above article had already been submitted for publication prior to its submission to *Molecular Medicine Reports*, the Editor has decided that this paper should be retracted from the Journal. After having been in contact with the authors, they accepted the decision to retract the paper. The Editor apologizes to the readership for any inconvenience caused.

